# Efficacy of Transdermal Ketoprofen Patch in Comparison to Transdermal Diclofenac Patch in Postoperative Analgesia for Orthodontic Extractions: A Randomized Split-Mouth Study

**DOI:** 10.7759/cureus.37732

**Published:** 2023-04-17

**Authors:** Kritika Pandey, Vijaylaxmi Shettar, Tejraj Kale

**Affiliations:** 1 Oral and Maxillofacial Surgery, Karnataka Lingayat Education (KLE) Vishwanath Katti Institute of Dental Sciences and Hospital, Belagavi, IND

**Keywords:** analgesic, ketoprofen, diclofenac, transdermal patch, orthodontic extractions

## Abstract

Introduction

Non-steroidal anti-inflammatory drugs (NSAIDs) are the most prescribed analgesics for controlling post-exodontia pain, administered by various routes. The transdermal route possesses the advantages of providing sustained release of the drug, being non-invasive, bypassing first-pass metabolism, and eliminating gastrointestinal adverse effects. This study compared the analgesic efficacy of diclofenac 200 mg and ketoprofen 30 mg transdermal patches for post-orthodontic exodontia pain.

Materials and methods

Thirty patients who underwent orthodontic bilateral maxillary and/or mandibular premolar extractions under local anaesthesia were included in the study. Each patient received single transdermal diclofenac 200 mg patch and single transdermal ketoprofen 30 mg patch on the outer, ipsilateral upper arm immediately post-extraction in the two appointments in random order. The pain score was recorded every second hourly for the first 24 hours postoperatively using a visual analog scale (VAS). The requirement of rescue analgesics at various time points and the total number of rescue analgesics taken in the first 24 hours postoperatively were noted. Any allergic reaction to the transdermal patches was also recorded.

Results

The analgesic efficacy of the two transdermal patches at any given time point in 24 hours by Mann-Whitney U test showed no statistically significant (p<0.05) difference. An overall intragroup statistically significant difference (p<0.05), by Wilcoxon matched pairs test, was found by comparison of VAS pain scores at different time points to that at 0-2 hours after application of transdermal ketoprofen and diclofenac patches, respectively. The mean maximum pain intensity was slightly lower for ketoprofen (2.33) than diclofenac (2.60) transdermal patch. Patients consumed the rescue analgesic within the first 12 hours postoperatively, with the mean value of the total number of rescue analgesics taken with ketoprofen transdermal patch (0.23) slightly lower than diclofenac transdermal patch (0.27) application.

Conclusion

Ketoprofen and diclofenac transdermal patches provide similar analgesia post orthodontic extraction. The patients required rescue analgesics only during the initial hours of the postoperative follow-up period.

## Introduction

Pain after minor or major oral surgical procedures is one of the actively studied subjects in pharmacology and pain research [1]. Monheim has defined pain as "an unpleasant emotional experience usually initiated by a noxious stimulus and transmitted over a specialized neural network to the central nervous system where it is interpreted as such" [2]. Studies have shown that sensory nociception in the oral cavity is significantly more than in many other areas of the body [1]. A normal atraumatic tooth extraction, once the effect of the local anesthetic wears off, may cause mild-to-severe pain, depending on the condition of the tooth and the patient's perception. Production of prostaglandins at the extraction site causes post-exodontia pain which is an uncomfortable experience for the patients that may lead to disruption of daily routine activities and may distract the individual from essential obligations [3]. Hence, measures should be taken to provide appropriate analgesia for the patient's uneventful post-exodontia healing phase [4]. Administration of either peripherally acting (non-steroidal anti-inflammatory drugs [NSAIDs]) or centrally acting (opioids) drugs is used for achieving postoperative analgesia. However, most post-exodontia pain is managed by NSAIDs worldwide [5]. One of the commonly prescribed NSAIDs for post-exodontia pain relief is diclofenac. Diclofenac is a non-selective cyclooxygenase enzyme inhibitor of the aryl acetic acid group that shows analgesic, anti-inflammatory, and antipyretic activity [6]. Diclofenac sodium acts by potent cycle-oxygenase inhibition, reduction of arachidonic acid release, and enhancement of arachidonic acid uptake. It thereby has a dual inhibitory effect on the cyclooxygenase and lipoxygenase pathways [7]. Ketoprofen, (RS)2-(3-benzoyl phenyl)-propionic acid, is one of the propionic acid classes of NSAIDs and has analgesic, anti-inflammatory, and antipyretic effects [8]. Ketoprofen combines with the cyclooxygenase, thereby preventing its substrate-enzyme combination with arachidonic acid and forming eicosanoids, prostaglandins, and thromboxanes. Ketoprofen also stabilizes lysosomes and inhibits lipoxygenase [5]. Ketoprofen has been well-documented in various studies as an effective agent in providing postoperative analgesia [8].

These drugs can be administered using various routes of drug administration, such as oral, IV, intramuscular (IM), sublingual, and transdermal [5]. The oral route of administration of drugs has been the route of choice in regular practice for a long time. However, this route poses the disadvantages of low bioavailability due to its first-pass metabolism, gastrointestinal adverse effects, and patient non-compliance. Other routes of administration of drugs commonly used in cases of extreme post-exodontia pain are IM or IV. IM injections of NSAIDs can lead to erythema, pruritis, edema, abscess, and necrosis. The IV route provides the fastest analgesia, but when the entire dose is injected within a short span, it can lead to systemic toxicity [9]. To overcome the drawbacks of the most commonly used routes of drug administration, a newer drug delivery agent, a transdermal patch, may be used by which the drug enters into systemic circulation through the skin or mucosa. Transdermal drug delivery systems are simple and compliant methods [10]. Transdermal patches possess the advantage of providing sustained release of the drug, a non-invasive method of drug administration. They bypass first-pass metabolism and eliminate gastrointestinal adverse effects [4]. In the present study, the efficacy of a single application of a transdermal ketoprofen patch versus a transdermal diclofenac patch in patients undergoing orthodontic extractions was compared for pain and the need for rescue analgesic over 24 hours postoperatively.

## Materials and methods

Study design and study setting

The study was carried out as a randomized split-mouth study in the Department of Oral and Maxillofacial Surgery, Karnataka Lingayat Education (KLE) Vishwanath Katti Institute of Dental Sciences and Hospital, Belagavi, Karnataka, India, for over 17 months from June 2021 to October 2022 following the principles of the Declaration of Helsinki. The ethical clearance was obtained from the Institutional Research and Ethics Committee with reference approval number 1467, dated May 5, 2021. Written informed consent was obtained from all the study patients.

Study population and sample size

A total of 30 patients, referred from the Department of Orthodontics and Dentofacial Orthopaedics with the requirement of bilateral maxillary premolar extractions and/or bilateral mandibular premolar extractions (therapeutic extraction of single premolar tooth ipsilaterally per arch) for orthodontic intervention, were included in the study who met the inclusion and exclusion criteria. The sample size for the study was calculated using the GPower program (G * Power version 3.1.9.4 statistical software) based on a previous study showing a comparison between the two transdermal patches in orthodontic extractions as a split-mouth study [4]. The sample size projections were made at 99% confidence levels (α error 0.01) and 95% power (β error 0.05).

Inclusion criteria

Patients aged 15 to 30 years requiring bilateral maxillary and/or mandibular premolar extraction for orthodontic intervention who reported to the Department of Oral and Maxillofacial Surgery with no known history of allergy to NSAIDs and willing to give written informed consent for the study were included in the study.

Exclusion criteria

Those patients with the presence of systemic disease(s), history of any drug intake pre-operatively, history of any deleterious such as tobacco chewing or smoking, and premolars associated with periodontal and periapical pathologic features were excluded.

Procedure

Assigned patients underwent extractions in the Department of Oral and Maxillofacial Surgery. A person not involved in the study was asked to pick the chit to randomly allocate the side of extraction and the transdermal patch to be applied in the first appointment. Patients underwent atraumatic extraction of the ipsilateral maxillary and/or mandibular first premolar(s) in the first appointment under local anesthesia. In the second appointment, 5-7 days later, patients underwent premolar(s) extraction on the contralateral side. A CONSORT flow diagram to demonstrate the enrollment and allocation is provided (Appendix 1). Extraction of teeth was carried out by conventional forceps method after administering regional anesthesia by infiltrating or blocking the nerve with 2% lignocaine plus adrenaline 1:80,000. A pressure pack was placed on the extraction site without socket approximation, and post-extraction instructions were given. Each patient received a commercially available single transdermal diclofenac 200 mg patch or transdermal ketoprofen 30 mg patch on the outer, ipsilateral upper arm immediately post-extraction. The transdermal patch to be applied in the first appointment was decided in random order, as mentioned earlier. Patients who received a diclofenac patch in the first appointment were given a ketoprofen patch in the second. In contrast, the patients who received the ketoprofen patch in the first appointment were given a diclofenac patch in the second. The patients were blinded to the analgesic patch being administered. The study patients were advised to take one rescue analgesic tablet (Tablet ketorolac 10 mg) if there was unbearable pain at any time during the first 24 hours post-extraction. The patient's postoperative pain was assessed using a 10-point visual analog scale (VAS) [11], recorded every second hour in the patient assessment sheet (Appendix 2). Follow-up was done 24 hours after the extraction to evaluate pain, adverse reactions, if any, associated with the patch, and the need for rescue analgesic within 24 hours after the atraumatic extraction.

Statistical analysis

Statistical analysis was done using SPSS software 20.0. Statistical significance was determined using the Mann-Whitney U and Wilcoxon matched pairs tests.

## Results

**Figure 1:**
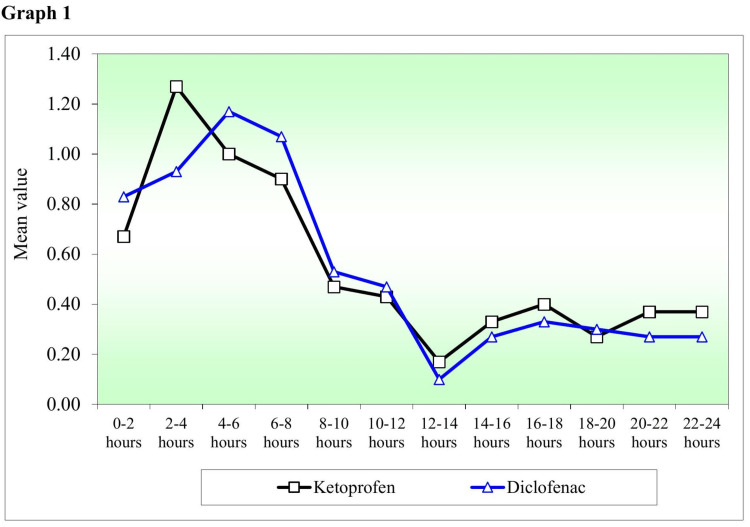
Comparison of mean VAS pain scores at different treatment time points after the application of transdermal ketoprofen patches and transdermal diclofenac patches. VAS: Visual analog scale.

In the present study, 30 patients (15 females, 15 males) aged 18.57 ± 2.53 years were included who required post-extraction analgesia after orthodontic bilateral maxillary and/or mandibular premolars extractions under local anaesthesia. Of the included patients, twenty-eight had Angle's Class II malocclusion, and two had Angle's Class I malocclusion with proclined maxillary incisors. Sixteen patients were administered transdermal ketoprofen 30 mg patch given in the first appointment and transdermal diclofenac 200 mg patch given in the second appointment. In comparison, 14 patients were administered transdermal diclofenac 200 mg patch given in the first appointment and transdermal ketoprofen 30 mg patch given in the second appointment. Figure 1 shows a graph that compares VAS pain scores at different treatment time points after applying transdermal ketoprofen patches and transdermal diclofenac patches. No statistically significant (p<0.05) difference was found between the analgesic efficacy of two transdermal patches at any given time point in 24 hours by the Mann-Whitney U test.

Table 1 demonstrates an intragroup comparison of VAS pain scores at different time points with VAS pain scores at 0-2 hours after applying transdermal ketoprofen and diclofenac patches, respectively, to the study patients. A statistically significant difference (p<0.05) in VAS pain score was found by Wilcoxon matched pairs test with ketoprofen patch at time points 2-4 hours, 12-14 hours, 14-16 hours, 18-20 hours as compared to VAS pain score at 0-2 hours; and with diclofenac group at time points 12-14 hours, 14-16 hours, 18-20 hours, 20-22 hours, 22-24 hours as compared to VAS pain score at 0-2 hours. Overall within each group, a statistically significant (p<0.05) difference was found between the VAS pain scores at different time points compared to the VAS pain scores at 0-2 hours.

**Table 1:** Intragroup comparison of VAS pain scores at different time points with VAS pain scores at 0-2 hours after applying transdermal ketoprofen and diclofenac patches. VAS: Visual analog scale.

Group	Changes from 0-2 hours to	Mean Diff.	SD Diff.	% of changes	Z-value	P-value	Friedman’s test	P-value
Ketoprofen	2-4 hours	-0.60	1.38	-90.00	2.2737	0.0230*	54.1856	0.0001*
	4-6 hours	-0.33	1.71	-50.00	0.8790	0.3794		
	6-8 hours	-0.23	1.76	-35.00	0.5491	0.5829		
	8-10 hours	0.20	1.40	30.00	0.8736	0.3824		
	10-12 hours	0.23	1.04	35.00	1.2230	0.2213		
	12-14 hours	0.50	0.68	75.00	3.0594	0.0022*		
	14-16 hours	0.33	1.03	50.00	2.2713	0.0231*		
	16-18 hours	0.27	1.26	40.00	1.3278	0.1842		
	18-20 hours	0.40	1.38	60.00	2.1181	0.0342*		
	20-22 hours	0.30	1.39	45.00	1.6449	0.1000		
	22-24 hours	0.30	1.47	45.00	1.6449	0.1000		
Diclofenac	2-4 hours	-0.10	1.30	-12.00	0.9124	0.3615	103.4617	0.0001*
	4-6 hours	-0.33	2.09	-40.00	0.8235	0.4102		
	6-8 hours	-0.23	2.42	-28.00	0.3314	0.7404		
	8-10 hours	0.30	1.73	36.00	0.7533	0.4513		
	10-12 hours	0.37	1.69	44.00	1.1531	0.2489		
	12-14 hours	0.73	1.39	88.00	2.5858	0.0097*		
	14-16 hours	0.57	1.81	68.00	2.1658	0.0303*		
	16-18 hours	0.50	1.91	60.00	1.7577	0.0788		
	18-20 hours	0.53	1.80	64.00	2.1344	0.0328*		
	20-22 hours	0.57	1.63	68.00	2.1573	0.0310*		
	22-24 hours	0.57	1.55	68.00	2.1315	0.0330*		

**Figure 2:**
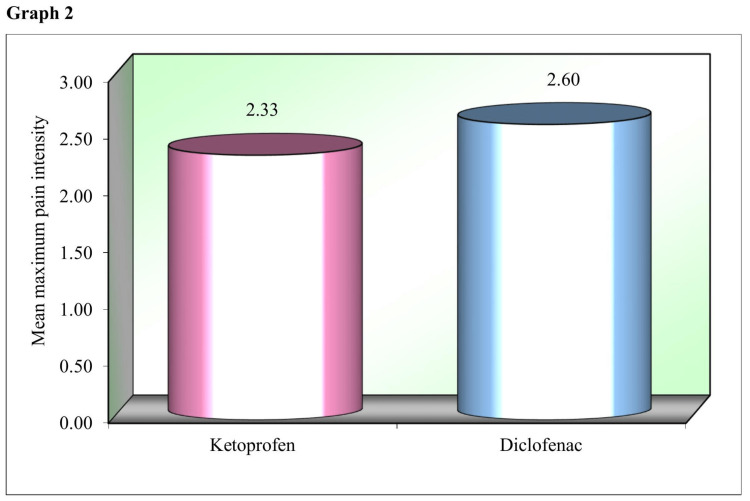
Comparison of the mean of maximum VAS pain score or maximum pain intensity experienced within the first 24 hours postoperatively after the application of ketoprofen and diclofenac transdermal patches. VAS: Visual analog scale.

Graph 2 (Figure 2) compares the mean of maximum VAS pain score or maximum pain intensity experienced by the study patients within the first 24 hours postoperatively after applying ketoprofen and diclofenac transdermal patches. The study patients had mean maximum pain intensity of 2.33 after the ketoprofen transdermal patch application, which was slightly lower than the mean maximum pain intensity of 2.60 after the diclofenac transdermal patch.

**Figure 3:**
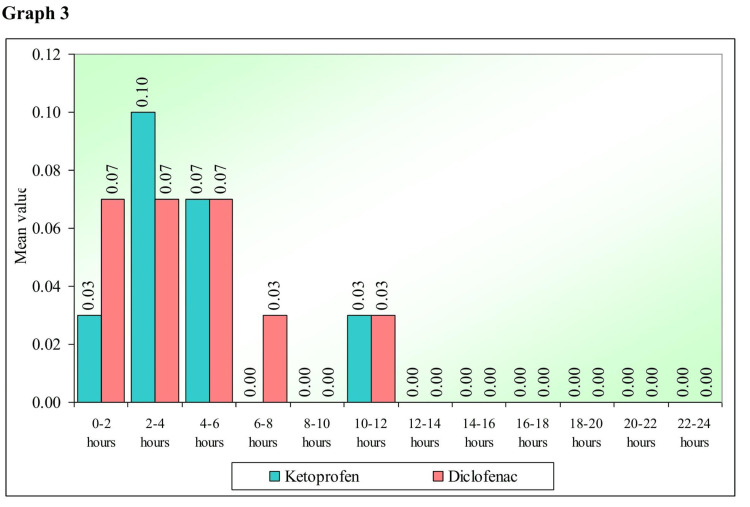
Comparison of the mean of rescue analgesics taken at different time points with ketoprofen and diclofenac transdermal analgesic patches.

Graph 3 (Figure 3) demonstrates a comparison of the mean of rescue analgesics taken by the study patients at different time points with ketoprofen and diclofenac transdermal analgesic patches, respectively. The study patients who took the rescue analgesic consumed it within the first 12 hours postoperatively, with maximum intake in the ketoprofen group between two and four hours.

**Figure 4:**
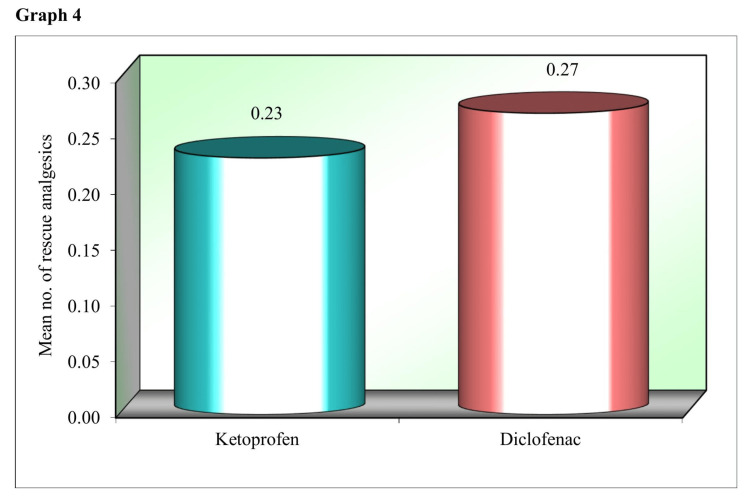
Comparison of the mean value of the total number of rescue analgesic intake in the first 24 hours post extraction after the application of transdermal ketoprofen and transdermal diclofenac patches.

Graph 4 (Figure 4) compares the mean value of the total number of rescue analgesic intake by the study patients in the first 24 hours post extraction after applying transdermal ketoprofen and transdermal diclofenac patches. The mean value of the total number of rescue analgesics taken with the ketoprofen transdermal patch is 0.23, which is slightly lower than the diclofenac transdermal patch (0.27) application.

## Discussion

Patients relate dental treatment with pain, especially minor oral surgical procedures performed under local anaesthesia. An eventful experience of poorly managed pain related to dental treatment can lead patients to avoid or delay treatment and make them apprehensive about future minor oral surgical procedures [12]. Therapeutic or orthodontic teeth extractions are often the first exodontia procedure for patients; hence, pain management is of utmost importance in such cases for removing fear instilled in patients related to minor oral surgical procedures. Post extraction, the medications are usually administered orally, but the oral route has certain disadvantages, such as low bioavailability due to its first-pass metabolism, gastrointestinal adverse effects, and patient non-compliance [8]. The transdermal drug delivery system, however, overcomes these problems. Transdermal therapeutic systems are devices that deliver the contained drug into systemic circulation by diffusion via the stratum corneum of the skin. The drug is held between an occlusive backing film and a rate-controlling micropore membrane as a reservoir. The micropore membrane is designed so that the rate of drug delivery to the skin surface is less than the slowest rate of absorption from the skin, eliminating any rate of absorption intra-site variability in an individual and inter-subject variability as well. Hence, a steady drug penetration across the dermis occurs, allowing for a consistent, uniform serum drug level and regulated drug absorption. The usual application sites are the chest, abdomen, upper arm, lower back, buttock, or mastoid region [5]. Pharmacokinetic data have suggested that transdermal NSAID patches have an increased tissue concentration to excrete a therapeutic effect but with plasma concentrations low enough, thus avoiding systemic complications. This drug delivery system also has enhanced bioavailability because it evades the first-pass hepatic metabolism and enzymatic or pH-associated deactivation associated with oral administration. Transdermal drug delivery is non-invasive, and hence it eliminates the fear of prick or injection associated with the invasive IM or IV route of injection [8]. Transdermal patch application has increased flexibility in terminating drug administration by patch removal [13]. Reported data, supported by the results from clinical trials, recommend that transdermal medications are equally efficacious compared with oral administration of the same drug. Transdermal patches provide effective postoperative analgesia for dentoalveolar surgical practice and major procedures, such as orthognathic, abdominal, and orthopedic surgeries [14]. Diclofenac is an analgesic, antipyretic, and an anti-inflammatory drug. It inhibits prostaglandin synthesis and is slightly COX-2 selective. Neutrophil chemotaxis and superoxide production at the inflammatory site is reduced if diclofenac is used. It is well absorbed orally, 99% protein bound, metabolized and excreted both in urine and bile. It has good tissue penetrability [5]. The plasma half-life of transdermal diclofenac is 12 hours as compared to 1.2-2 hours with oral diclofenac [15]. 

Ketoprofen (nonselective COX inhibitors, propionic acid derivative) is an analgesic, antipyretic, and anti-inflammatory drug. It inhibits prostaglandin synthesis, stabilizes lysosomes, and inhibits lipooxygenase. It is highly bound to plasma proteins (90-99%), enters the brain and synovial fluid, and crosses the placenta. They are largely metabolized in the liver by hydroxylation and glucuronide conjugation and excreted in urine and bile [5]. The plasma elimination half-life of oral ketoprofen is 2-4 hours [16]. The systemic absorption of transdermal ketoprofen is approximately 2 orders of magnitude less than that of oral ketoprofen [17]. Diclofenac and ketoprofen are preferred NSAIDs used in clinical practice for postoperative pain management. Selvi UG et al. [18] in 2016 compared transdermal diclofenac patch with intramuscular diclofenac injection as an analgesic modality following surgical extraction of impacted mandibular third molars in a cross-over efficacy trial and concluded that diclofenac administered by either mode of delivery has similar effectiveness. Bhaskar H et al. [19] in 2010 compared transdermal diclofenac patch with oral diclofenac as an analgesic modality following multiple premolar extractions in orthodontic patients. They concluded that both diclofenac formulations provided potent analgesia with the advantage of better patient compliance for the transdermal route. These studies concluded that patient compliance with the patch was greater than that with other routes of drug administration. Mazières B et al. [20] in 2005 tested the efficacy and tolerability of a 100 mg patch of ketoprofen applied once a day for treating ankle sprain and concluded that a seven-day course of treatment with ketoprofen patch is useful in benign ankle sprain without revealing unexpected adverse events. Osterwalder A et al. [21] in 2002 evaluated tissue absorption and distribution of ketoprofen after patch application in subjects undergoing knee arthroscopy or endoscopic carpal ligament release. They concluded that ketoprofen applied on the skin can enter the subcutaneous and intra-articular tissues, reaching concentrations significantly higher than in plasma to produce the desired pharmacological activity in situ, and plasma concentrations are low enough to avoid any systemic adverse or side effects. In the present study, 30 patients with a mean age of 18.57 ± 2.53 years who underwent orthodontic bilateral maxillary and/or mandibular premolars extractions under local anaesthesia were included and required post-extraction analgesia. The extractions were done in two separate appointments with a latency period of at least five days between the interventions. The study population comprised 15 males (50%) and 15 females (50%). The purpose of the present study was to compare the analgesic efficacy of transdermal ketoprofen and diclofenac patches for patients who had undergone atraumatic extraction of first premolars for orthodontic purposes. The evaluation was done based on the VAS pain score [11] recorded every second hour for the first 24 hours postoperatively, the requirement of rescue analgesics at various time points, and the total number of rescue analgesics taken in the first 24 hours postoperatively. Any allergic reaction or complications to using analgesic patches was also recorded. Rescue analgesic was prescribed to the patients as an alternative for analgesia if the transdermal patches did not provide sufficient pain relief at a particular time. Ketorolac 10 mg (nonselective COX inhibitors, acetic acid derivatives) is frequently used in postoperative, dental, and acute musculoskeletal moderate pain management for short-term [5], which is why in the present study as well the same drug was prescribed as a rescue analgesic.

The results of the present study have shown that the ketoprofen transdermal patch and diclofenac transdermal patch provides similar analgesia post-orthodontic extraction. Verma R et al. [8] in 2016 compared single-dose transdermal patches of diclofenac (100 mg) and ketoprofen (20 mg) for postoperative analgesia in lower limb orthopedic surgery. They concluded that transdermal patches of ketoprofen and diclofenac are both effective for postoperative analgesia in lower limb orthopedic surgery under spinal anaesthesia. However, in the diclofenac group, more patients required rescue analgesics than the ketoprofen group. Bhargava D et al. [4] in 2019 compared the efficacy of transdermal ketoprofen (20 mg) and diclofenac (200 mg) patches in patients undergoing therapeutic atraumatic first premolar extractions. They concluded that ketoprofen is superior to diclofenac as a transdermal medicament in providing post-extraction analgesia. No statistically significant (p<0.05) difference was found between the analgesic efficacy of two transdermal patches at any given time point in 24 hours. However, patients reported lower maximal pain intensity with ketoprofen transdermal patch application compared to diclofenac patch application, consistent with the above studies. The mean VAS score for maximum pain intensity was 2.33 for the ketoprofen patch compared with a mean VAS score for maximum pain intensity of 2.60 for the diclofenac patch. However, Bachalli PS et al. [12] in 2016 conducted a comparative study of a diclofenac transdermal patch (100 mg) against oral diclofenac (100 mg) for pain control following the removal of mandibular-impacted third molars. They concluded that transdermal diclofenac sodium can be used as an alternative form of pain control following the removal of impacted mandibular third molars, however considering that the analgesic potency might be lesser in the immediate postoperative period, it might be prudent to use oral diclofenac sodium for immediate postoperative pain relief, following which transdermal route can be used for pain control. This finding is consistent with our study, where patients had the highest VAS pain scores between two and six hours postoperatively for both the patches and consumption of the rescue analgesic was maximum during the same time period. In the present study, six patients took rescue analgesics when the ketoprofen patch was applied. In the same patients, when the diclofenac patch was applied on the contralateral side, seven patients took rescue analgesics. In total, only seven patients took rescue analgesics with both patches. The patients who took rescue medication consumed them between 0 hours and 10-12 hours postoperatively. No allergic or adverse reactions were reported to either of the patches among the study participants, which is similar to the available literature.

In general, according to the literature, ketoprofen has been found to be more potent than diclofenac, although the use of ketoprofen has not been popular in clinical practice. In our study, ketoprofen faired better in providing post-exodontia analgesia than diclofenac in terms of the maximum VAS score recorded by the study patients after each transdermal patch application. However, the difference between the VAS scores was not statistically significant at any of the different time points in 24 hours follow-up period. The study's strength is the use of the transdermal drug delivery system, which enters into systemic circulation through the skin. It overcomes the drawbacks of the most commonly used route of drug administration for analgesia after the exodontia procedure, i.e., oral. The oral route of drug administration has the disadvantages of low bioavailability due to its first-pass metabolism, inconsistent absorption, gastrointestinal adverse effects such as acidity and nausea, and patient non-compliance. Transdermal patches are compliant methods of drug delivery that bypass first-pass metabolism, thus providing drug delivery at a constant and predictable rate, smooth plasma concentrations of the drug without fluctuations, and, hence, nullifying inter-individual variations. They are non-invasive and have minimal side effects. So, in most cases, patients can altogether avoid oral medications and, eventually, the associated problems. The present study is a split-mouth randomized study that renders the removal of inter-subject variability, which is also its major strength. The major drawbacks of the study include the limited sample size, transdermal patches used in the study being costlier than commonly prescribed oral analgesics, and some patients requiring rescue analgesics in the initial few hours post-exodontia. The comparison of the effectiveness of transdermal analgesic patches in other minor and major surgical procedures with a larger sample size needs to be evaluated in future clinical studies to validate the results of the present study.

## Conclusions

Transdermal drug delivery systems are simple and compliant methods of delivery. They possess the advantages of providing sustained drug release, a non-invasive method of drug administration, bypassing first-pass metabolism, and eliminating gastrointestinal adverse effects. With the results of the current study, we conclude that both ketoprofen and diclofenac transdermal patches were effective for postoperative analgesia after orthodontic extraction under local anaesthesia, with the maximum pain intensity being lower with the ketoprofen transdermal patch. The patients in the current study who required rescue analgesics consumed them during the initial hours of the postoperative follow-up period, after which they were comfortable. We infer that the transdermal ketoprofen and diclofenac analgesic patches, after application, achieve their clinical effectiveness after a time-lapse of around four-to-six hours. So, patients require other routes of drug administration for immediate postoperative pain relief. Hence, studies can be done in the future to evaluate these transdermal patches as a pre-emptive measure for pain relief so that a therapeutic plasma concentration of the drug is attained in the initial postoperative period itself for good pain control throughout the follow-up period.

## Figures and Tables

**Figure 5:**
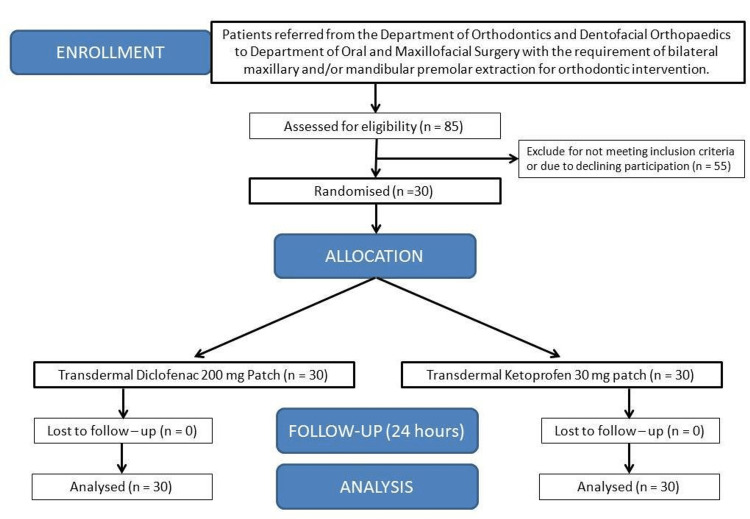
Consolidated Standards of Reporting Trials (CONSORT) diagram.

**Figure 6:**
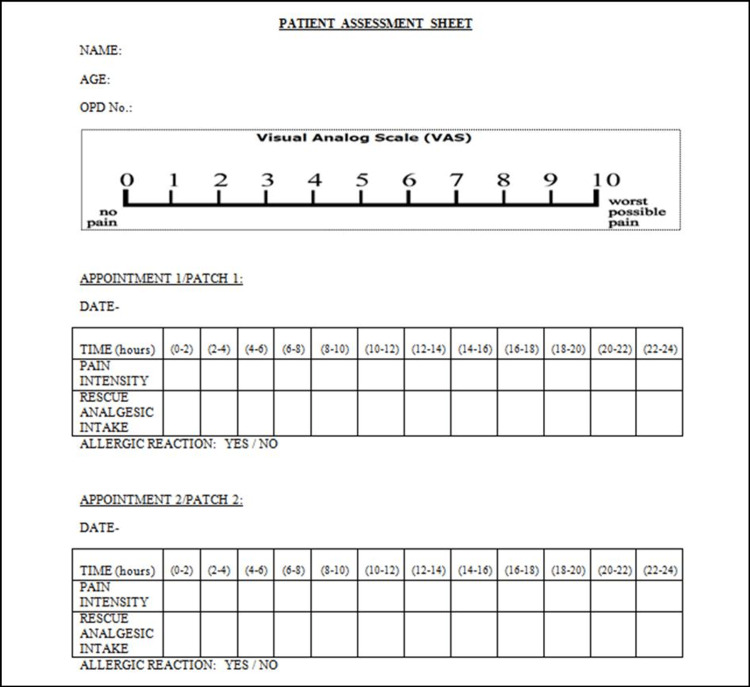
Patient assessment sheet.

## Appendices

Appendix 1

Appendix 2 
